# Quantitative sensory testing as an assessment tool to predict the response to standard pain treatment in knee osteoarthritis: a systematic review and meta-analysis

**DOI:** 10.1097/PR9.0000000000001079

**Published:** 2023-06-05

**Authors:** Kristian Kjær-Staal Petersen, Kübra Kilic, Emma Hertel, Trine Hyttel Sejersgaard-Jacobsen, Marlene Kanstrup Jørgensen, Anders Troelsen, Lars Arendt-Nielsen, Dennis Boye Larsen

**Affiliations:** a Center for Neuroplasticity and Pain (CNAP), SMI, Department of Health Science and Technology, School of Medicine, Aalborg University, Aalborg, Denmark; b Center for Mathematical Modeling of Knee Osteoarthritis (MathKOA), Department of Material and Production, Faculty of Engineering and Science, Aalborg University, Aalborg, Denmark; c Department of Anesthesia, Aalborg University Hospital, Thisted, Denmark; d Department of Orthopedic Anesthesia, Aalborg University Hospital, Aalborg, Denmark; e Department of Orthopedic Surgery, Copenhagen University Hospital, Hvidovre, Denmark; f Department of Medical Gastroenterology, Mech-Sense, Aalborg University Hospital, Aalborg, Denmark

**Keywords:** Osteoarthritis, Quantitative sensory testing, Total knee arthroplasty, Nonsteroidal anti-inflammatory drugs, Exercise-based therapies

## Abstract

Emerging evidence suggest that quantitative sensory testing (QST) may predict the treatment response to pain-relieving therapies. This systematic review and meta-analysis focus on the predictive value of QST for pain management of knee osteoarthritis (OA). MEDLINE and EMBASE were systematically searched for all studies from year 2000 to 2023 on pretreatment QST and treatment of OA including surgical, pharmaceutical, and nonsurgical and nonpharmaceutical therapies. Preclinical studies and reviews were excluded. The systematic review followed the PRISMA guidelines and was pre-registered on the Open Science Framework website (link: https://osf.io/4FETK/, Identifier: DOI 10.17605/OSF.IO/4FETK). Meta-analysis were conducted to demonstrate the strength of the pre-treatment QST predictions on pain outcomes after OA treatments. Sixteen surgical (all on total knee arthroplasty [TKA], N = 1967), 5 pharmaceutical (4 on non-steroidal anti-inflammatory drugs [NSAIDs], N = 271), and 4 exercise-based therapy studies (N = 232) were identified. Pretreatment QST parameters predicted pain-relieving treatment outcomes in 81% of surgical, 100% of pharmaceutical, and 50% of exercise-based therapy studies. Meta-analyses found pretreatment QST profiles to predicted pain outcomes after TKA (random effects: 0.309, 95% confidence interval [CI]: 0.206–0.405, *P* < 0.001), NSAIDs (random effects: 0.323, 95% CI: 0.194–0.441, *P* < 0.001), and exercise-based therapies (random effects: 0.417, 95% CI: 0.138–0.635, *P* = 0.004). The overall risk of bias for the included studies was low to moderate. This systematic review and meta-analysis demonstrate weak-to-moderate associations between pretreatment QST and pain outcomes after standard OA pain treatments. Based on this work, it is hypothesized that a subset of specific pain sensitive patients with OA exist and that these patients do not respond adequately to standard OA pain treatments.

## Abstract 2

Supplemental Digital Content is Available in the Text.

This work indicates that a subset of specific pain sensitive patients with osteoarthritis exists and that these patients do not respond adequately to standard pain treatments.

## 1. Introduction

Osteoarthritis (OA) is a major clinical problem with an estimated prevalence of 3754 per 100,000,^53^ and the prevalence is expected to increase in the future.^11,13,22^ Osteoarthritis Research Society International (OARSI) provides recommendations for the treatment of pain in OA^9,66^ with (1) surgical, (2) pharmaceutical therapy, and (3) exercise-based therapy in combination with patient education being the most common. In addition, total knee arthroplasty (TKA), topical and oral nonsteroidal anti-inflammatory drugs (NSAIDs), and exercise-based therapy in combination with patient education is considered the standard pain therapies for the treatment of pain in OA, and more recently, duloxetine have been conditionally recommended as a treatment option for a subset of patients with OA pain.^10^ It is well described that these treatments provide patients with pain relief but that a subpopulation of patients does not obtain substantial effects of the treatments.^12,17,57^ Methods to identify patients in risk of a poor response before these treatments could improve health care and potentially lead to personalized pain medicine.

Quantitative sensory testing (QST) has been suggested as surrogate measure for peripheral and central pain mechanisms.^7^ In particular, pressure pain thresholds (PPT), temporal summation of pain (TSP), and conditioned pain modulation (CPM) are often used to profile patients with OA.^2^ Lower PPTs assessed over a painful area (eg, a painful OA knee) mainly reflect localized hyperalgesia, whereas lower PPTs assessed outside of a painful area reflect widespread pressure hyperalgesia, which, based on animal studies, is considered a sign of central pain sensitization.^1^ Temporal summation of pain is considered a proxy for the phenomenon of wind-up in dorsal horn neurons where the same stimulus is applied several times at fixed intervals and intensities yielding increased pain perception.^25^ Conditioned pain modulation is assumed to be the human surrogate model for diffuse noxious inhibitory controls assessed in animals,^36^ reflecting the balance of descending pain inhibitory and facilitatory mechanisms.^62^ In general, patients with severe OA exhibit lower PPTs (locally and widespread), facilitated TSP, and impaired CPM when compared with healthy asymptomatic subjects,^4^ and emerging evidence suggest that QST might be a predictive tool for standard pain treatments.^49^ Studies indicate that some patients are more pain sensitive than others,^3,23,31^ and the new pain descriptor “nociplastic” may apply to these pain sensitive patients.^33^

Parades et al.^42^ reviewed the literature on the predictive role of QST on acute and chronic pain after TKA and identified 9 studies in which preoperative QST predicted chronic postoperative pain, but the field has grown since the Parades et al.^42^ review, and it is currently unknown if these predictions can be applied to other standard pain therapies for OA as recommended by the OARSI.^9^ The current paper aims to provide an up-to-date systematic review and meta-analysis on the possible role of specific QST parameters to predict outcome after surgical, pharmacological, and exercise-based therapies in OA.

## 2. Methods

In accordance with the Preferred Reporting Items for Systematic Reviews and Meta-Analyses (PRISMA) statement, this systematic review investigated the predictive role of QST on standard pain therapies for knee OA. The systematic review followed the PRISMA guidelines and was registered on the Open Science Framework website (OSF.IO, registration link: https://osf.io/4FETK/, study identifier: DOI: 10.17605/OSF.IO/4FETK). Studies from year 2000 to 2022 were included.

### 2.1. Search strategy and selection of studies

A systematic literature search was performed in February 2023 in the databases MEDLINE and EMBASE by 2 reviewers (K.K.P. and D.B.L.).

An example of the MeSH terms and text words used in each database is provided in Supplementary table 1 and 2 (available at http://links.lww.com/PR9/A194). The reference manager Mendeley was used to export the citations, and all the duplicates were excluded.

### 2.2. Eligibility criteria

Studies were included if they investigated one or more preoperative QST measures, including thermal, pressure, electrical, mechanical pain detection, tolerance, and suprathreshold stimuli, TSP, CPM, or exercise-induced hypoalgesia before standard pain treatment for OA. Furthermore, studies had to investigate associations between preoperative QST measures and the pain-related outcome after surgery, pharmaceutical therapies, or nonsurgical and nonpharmaceutical therapies by means of correlations (Spearman and Pearson correlations), regression models, or other predictive models.

A minimum of 6 months postoperative follow-up was chosen for surgical studies to assess chronic pain, as earlier research has reported the largest pain improvement 3 to 6 months after, eg, total knee arthroplasty surgery.^61^ Pharmacological and exercise-based studies were included if they investigated long-term effects of therapy (weeks/months), whereas studies focused on the acute effects of pharmacological or exercise-based therapies were excluded. In addition, the exclusion criteria consisted of languages other than English, conference abstracts, and animal studies.

Pain outcomes were reported through pain intensity, postoperative pain relief, presence of moderate-to-severe postoperative pain, or validated questionnaires on pain and disability including the Western Ontario and McMaster Universities Osteoarthritis Index (WOMAC), Visual Analogue Scale (VAS), and Numerical Rating Scale (NRS).

### 2.3. Data extraction and synthesis of included literature

The title of publications identified in the databases was reviewed by 2 reviewers (K.K.P. and D.B.L.) in a blinded fashion before meeting. After removing duplicates, abstracts of the articles were screened for potential eligibility and posterior full-read text by the same 2 reviewers (K.K.P. and D.B.L.) independently. The data were independently extracted by 2 investigators (K.K.P. and D.B.L.). For each study, the recorded data were on the total number of subjects, the pretreatment predictors (including QST paradigms), the follow-up time, the dependent outcome of the predictive model, and type of the predictive model. In case of discrepancies in data extraction and synthesis, a third investigator (LAN) was available to make the final decisions.

### 2.4. Quality assessment

Quality In Prognostic Studies (QUIPS) tool was independently used by 2 authors (K.K.P. and D.B.L.) to assess the quality and the methods of the included articles, more specifically to assess the overall risk of bias in each study focusing on 6 bias domains: study participation, study attrition, prognostic factor measurement, outcome measurement, study confounding, and statistical analysis and reporting. If consensus was not reached, a third independent reviewer (LAN) was consulted for the final decision.

### 2.5. Meta-analysis

The studies identified for the systematic review displayed a large degree of heterogeneity in the reporting outcomes, which complicated a traditional meta-analysis. To overcome this, studies reporting only compound *R*^2^ values (predictive models) or odds ratios were transformed to Pearson correlation coefficients (*r*) for the meta-analysis. This was performed to ensure the same association statistic was used for an overall correlation coefficient, at the expense of limiting the ability to infer whether the correlation is mainly driven by the QST measure or other preintervention factors in the case of compound statistics. Where possible, single correlations between preoperative QST measures and pain outcome(s) were preferred and included in the overall meta-analysis instead of compound correlations. Here, it is important to delineate the extent to which pain sensitive QST profiles were considered with respect to pain outcome. The meta-analysis was performed to investigate if signs of pain sensitization, as reflected by QST proxies, were associated with treatment outcomes. The reverse sign correlation was used for PPT and CPM data^34,35,41^ so that a positive correlation indicated a pain sensitive profile. Correlations based on compound predictive models would be expected to have positive correlations (as the *R*^2^ cannot be negative). Forest plots were generated to exhibit the correlational strengths between preoperative QST measures and treatment outcomes and further highlight if studies reported correlations based on compound models or singular association statistics. A higher value on the forest plot indicates a stronger association between a pretreatment QST, whereas positive values indicate that pain sensitive (pronociceptive) subjects are more likely for a poor pain-relieving outcome after therapy, and a negative value indicate that a less pain sensitive (antinociceptive) subject is more likely for a poor pain-relieving outcome after therapy.

The meta-analysis was conducted using MedCalc (v. 20.103; MedCalc Software Ltd, Ostend, Belgium), applying the Fisher *z* transformation of correlation coefficients and Hedges–Olkin method for weighted summary correlation coefficients under a fixed effect model. As the meta-analysis is conducted on the Fisher *z* transformed values, data were pooled independent if correlations were based on relative^6,45,58^ or absolute^14,16,21,34,35,38,44,46,50,52,59^ pain outcomes. Studies with multiple QST outcomes with an eligible correlation to treatment outcome^6,34,41,58^ were still included with both QST parameters, but halved in population per the Cochrane guidelines, to avoid double counting.^29^ Since heterogeneity is assumed for the included studies due to, eg, methodological differences, the summed correlation coefficient between the QST measures and pain outcome after surgery, analgesic treatment, or exercise, was estimated and plotted by both the fixed-effects and the random-effects model. Heterogeneity was assessed using the χ^2^ test and I^2^ statistics, where a χ^2^ test *P* < 0.1 suggests significant heterogeneity with I^2^ > 60% reflecting substantial heterogeneity.^29^

## 3. Results

**Figure 1.**
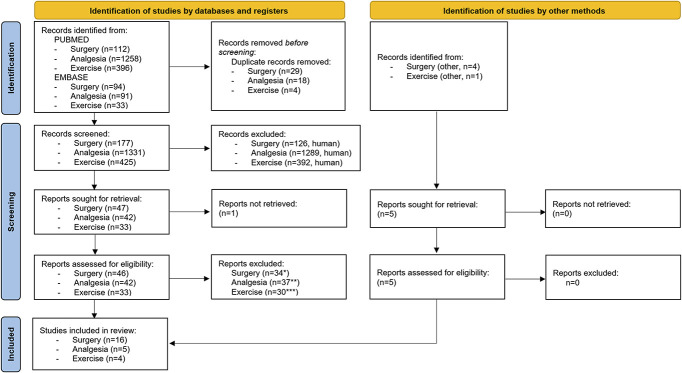
The Preferred Reporting Items for Systematic Reviews and Meta-Analyses (PRISMA) flow diagram. Reason(s) for exclusion: *Not postoperative period/pain (n = 9), not total knee arthroplasty (n = 1), no QST measure (n = 4), no prediction for postoperative pain (n = 2), abstract (n = 2), review or commentary (n = 7), protocol (n = 2), and duplicate not caught by automated duplicate procedure (n = 1); **Not postoperative period/pain (n = 3), not osteoarthritis population (n = 3), no QST measure (n = 2), no prediction for analgesic effect (n = 14), abstract (n = 2), review or commentary (n = 8), protocol (n = 2), duplicate not caught by automated duplicate procedure (n = 1), and not standard treatment (n = 1); ***no pain sensory profiling measure (n = 15), no prediction for exercise effect on pain (n = 5), abstract (n = 3), review or commentary (n = 2), protocol (n = 1), only acute effects (n = 1), no prediction for postexercise pain (n = 2), and secondary analysis of data already included (n = 1). QST, quantitative sensory testing.

Two independent investigators (K.K.P. and D.B.L.) screened 1933 publications by title and abstract to exclude articles that did not meet the inclusion criteria. By consensus between D.B.L. and K.K.P., the initial included articles were decided for 90% of records. One senior investigator (L.A.N.) was consulted for final decisions on the remaining articles, and consensus was reached for all articles. The PRISMA flow diagram (Fig. 1) illustrates the search process, where 25 eligible publications were identified through the systematic literature search.

A total of 25 studies were identified where 16 studies focused on surgery (all on total knee arthroplasty, Table 1), 5 studies on pharmacological treatments (Table 2), and 4 studies investigated exercise-based therapy or exercise-based therapy in combination with patient education (Table 2). Sample sizes ranged from 14 to 288 with a total of 2238 patients (1967 patients in the surgical studies, 271 patients in the pharmacological studies, and 232 patients in the nonsurgical and nonpharmacological studies).

**Table 1:** Studies assessing preoperative quantitative sensory testing (QST) as a predictor for chronic postoperative pain 3 months or longer in patients undergoing total knee arthroplasty.

Reference	Year	Patients (N)	QST	Follow-up (mo)	POP outcome	Preoperative findings
Lundblad et al.^38^	2008	69	EDT and EPT	18	VAS	Regression (M): Preop pain (OR) = 6.48 EPT (OR) = 9.19
Wylde et al.^59^	2013	51	PPT and HPT	12	WOMAC	PPT; correlation (U) *r* = 0.37, (*R*^2^ = 0.1369)
Noiseux et al.^40^	2014	193	MPT_,_ HPT, and PPT	6	Moderate-to-severe postoperative pain (NRS)	Regression (M): No predictive value
Petersen et al.^44^	2015	78	PPTs and TSP CPM	12	VAS	Regression (M): TSP and preop VAS: *R*^2^ = 0.13*
Wylde et al.^60^	2015	239	PPT	12	WOMAC	No predictive value
Petersen et al.^45^	2016	103	PPT, PTT_,_ TSP, and CPM	12	VAS	Regression (M): *R*^2^ = 0.379, using PPT and VAS* Combined facilitated TSP/impaired CPM associated with less pain relief
Vaegter et al.^58^	2017	14	PPTs_,_ PTT_,_ CPM, and EIH	6	NRS	Correlations (U): CPM *r* = 0.57 (*R*^2^ = 0.3249) EIH correlation *r* = 0.53 (*R*^2^ = 0.2809)
Bossmann et al.^14^	2017	47	TSP and CPM	6	WOMAC	Regression (M): No predictive value
Arendt-Nielsen et al.^6^	2018	70	PPT	12	No pain (VAS 0–4 mm)	Correlation (U): PPT affected limb (*R*^2^ = 0.110), PPT contralateral limb (*R*^2^ = 0.09)
Petersen et al.^46^	2018	130	HPT, WDT, CDT, CPT, and TSP	12	VAS	Regression (M): Preop mTSP, WDT, HPT, and KL: *R*^2^ = 0.119*
Rice et al.^52^	2018	288	TSP_,_ PPT_,_ and CPM	6 and 12	WOMAC	Regression (M): TSP: OR = 1.06, WOMAC: OR = 1.01 AUC: 0.70, specificity: 0.64, sensitivity: 0.72 65.67% correctly classified at 6 mo. No prediction at 12 mo
Kurien et al.^34^	2018	50	PPTs_,_ TSP_,_ PTT_,_ TSP_,_ and CPM	6	VAS	Correlation (U): PTT *r* = −0.262, (*R*^2^ = 0.0686); TSP *r* = 0.343 (*R*^2^ = 0.1176)
Larsen et al.^35^	2021	131	PTT, PPT_,_ and CPM	12	VAS	Correlation (U): CPM: *r* = −0.18 (*R*^2^ = 0.0324) CPM: MB-lin-reg-ana, β = −0.124, *P* = 0.122 contributed to the variance explanation but was not an independent factor
Dürsteler et al.^18^	2021	146	CPM	6	The presence of postoperative pain (NRS>3 at 6 mo postoperative follow-up)	CPM; correlation *P* = 0.004 Strength of correlation not reported
Bruehl et al.^16^	2022	110	TSP	6	CRPS CSS	Correlation (U): TSP *r* = 0.22
Edwards et al.^21^	2022	248	PPTs, TSP, and CPM	6	WOMAC BPI	Regression (M): TSP *r* = 0.316 (*R*^2^ = 0.10)

* Not reported in the original paper but calculated for this review.

ALL, allodynia; AUC, area under the receiver operating characteristic curve; CDT, cold detection threshold; CPM, conditioned pain modulation; CPT, cold pain threshold; CRPS, complex regional pain syndrome; CSS, CRPS severity score; EDT, electrical detection threshold; EIH, exercise-induced hypoalgesia; EPT, electrical pain threshold; HPT, heat pain threshold; lin-reg, linear regression; M, multivariate analysis; MB-lin-reg ana, multiple backward linear regression analysis; MPT, mechanical pain threshold; NRS, numeric rating scale; OR, odds ratio; *P*, probability value; POP, postoperative pain; PPT, pressure pain threshold; PTT, pain tolerance threshold; *r*, correlation coefficient; TSP, temporal summation of pain; U, univariate analysis; VAS, Visual Analog Scale; WDT, warm detection threshold; WOMAC, Western Ontario and McMaster Universities Osteoarthritis Index.

**Table 2:** Studies assessing pretreatment quantitative sensory testing (QST) as a predictor for pharmacological and exercise-based therapy studies.

Reference	Year	Patients (N)	Treatment	QST	Follow-up	Treatment outcome	Findings
Pharmacological treatments							
Arendt-Nielsen et al.^5^	2016	37	COX-2 inhibitor	PPT, TSP, and CPM	4 wk	Change in pain intensity for nonresponders	Correlation (U): *r* = 0.64 for nonresponders (less than 30% pain alleviation in 16 patients)
Edwards et al.^20^	2016	35	Topical NSAID (gel)	PPT, TSP, and CPM	4 wk	Change in average daily pain intensity (ADP) and KOOS pain	Regressions (M): ADP: CPM: *R* = −0.38
Petersen et al.^47^	2019	132	Oral NSAID and paracetamol	PPT, PTT, and TSP	3 wk	VAS (worst pain and during activity)	Regression (M): *R*^2^ = 0.24–0.27 using the VAS and TSP
Petersen et al.^48^	2019	42	Oral NSAID and paracetamol	CPM, offset analgesia	3 wk	VAS (worst pain and during activity)	Regression (M): *R*^2^ = 0.19 using the VAS and CPM
Petersen et al.^50^	2022	25	Duloxetine	PPT, PTT TSP, and CPM	16 wk	Change in BPI and WOMAC	Regression (M): *R*^2^ = 0.46–0.76 using TSP, PTT, BPI, WOMAC, and HADS
Exercise-based therapy							
Henriksen et al.^28^	2014	RCT: Exercise group: N = 31 Control: N = 29	3 group based or invidividual session per week	PPT and TSP	12 wk	Change in KOOS from baseline to follow-up	Correlation: No predictive value (change in PPTs from baseline to follow-up was associated with outcome: *R*^2^ = 0.35)
O'Leary et al.^41^	2018	99	4–6 group-based or individual sessions	PPT, TSP, CPM, MDT, VDT, and heat and cold hyperalgesia (thermal rolls)	10.5 wk (average)	Responders and nonresponders according to the OMERACT-OARSI responder criteria	Regression (U): OR for nonresponds: TSP: OR 2.00, 95% CI 1.23–3.27 PPT: 0.48 (95% CI 0.29–0.81)
Arendt-Nielsen et al.^6^	2018	49	Education, exercise, and insoles. Weight loss and pain medicine, if needed. Two sessions per week for 8 weeks and continued for to a total of 3 mo	PPT	12 mo	VAS after walking	Regression (M): No predictive value
Hansen et al.^27^	2020	24	12 sessions of neuromuscular exercises (twice weekly)	PPT, TSP, and EIH		Responders according to the OMERACT-OARSI responder criteria	Regression (M): *R*^2^ = 0.468 with PPT, EIH, and PDQ

BPI, Brief Pain Inventory; CPM, conditioned pain modulation; EIH, exercise-induced hypoalgesia; M, multivariate analysis; MDT, mechanical detection threshold; NRS, numeric rating scale; OR, odds ratio; *P*, probability value; PDQ, PainDetect Questionnaire; PPT, pressure pain threshold; PTT, pain tolerance threshold; *r*, correlation coefficient; TSP, temporal summation of pain; U, univariate analysis; VAS, Visual Analog Scale; VDT, vibration detection threshold; WDT, warm detection threshold; WOMAC, Western Ontario and McMaster Universities Osteoarthritis Index.

### 3.1. Quantitative sensory testing modalities utilized used in the studies

A total of 25 eligible publications were identified through the systematic literature search with 16 surgical, 5 pharmacological therapy, and 4 exercise-based therapy studies.

### 3.2. Surgical studies

#### 3.2.1. Electrical stimuli

Electrical stimuli were reported 1/15 studies (6%)^38^ as electrical detection and electrical pain thresholds (EPTs). Lundblad et al.^38^ demonstrated that lower preoperative EPTs in combination with higher preoperative pain intensity were predictive of the 18-month postoperative pain intensity.

#### 3.2.2. Pressure stimuli

Pressure stimuli were reported in 11/16 studies (69%),^6,21,34,35,40,44,45,52,58–60^ as assessed by pressure pain thresholds using cuff algometry (cPPT),^21,34,35,45,58^ handheld algometer (PPT),^6,21,34,40,44,52,58–60^ and pain tolerance thresholds using cuff algometry (cPTT).^34,35,45,58^ Pressure stimuli were predictive in 4/11 (36%) studies using cPDT,^45^ PPT,^6,59^ and cPTT.^34^

Wylde et al., 201^59^ found that preoperative lower PPTs assessed at the forearm were associated with the 1-year postoperative WOMAC score. Petersen et al.,^45^ found that lower preoperative cPPTs assessed at the lower leg were associated with lower levels of 12 months postoperative pain relief. Arendt-Nielsen et al.,^6^ found that lower preoperative PPTs assessed at the affected and nonaffected limb was associated with pain intensity after walking change from baseline to 12 months. Kurien et al., 2018 found that lower preoperative cPTT was associated with postoperative pain intensity at 6 months.

#### 3.2.3. Thermal stimuli

Thermal stimuli were reported in 4/16 studies (25%)^40,46,59^ and found predictive of chronic postoperative pain in one study (25%).^46^ Cold detection threshold (CDT),^46^ and warm detection threshold (WDT)^46^ were assessed in one study, cold pain threshold (CPT)^21,39,46^ were assessed in 3 studies, whereas heat pain threshold (HPT) was assessed in 3 studies.^40,46,59^ Petersen et al.,^46^ found that lower HPT and lower WDT in combination with higher TSP and lower Kellgren and Lawrence scores were predictive for chronic postoperative pain intensity.

#### 3.2.4. Temporal summation of pain

Temporal summation of pain was reported as the QST parameter in 8/16 (50%) studies^14,16,21,34,44–46,52^ as assessed using cuff algometry (TSP_cuff_)^34,45^ or monofilaments (mTSP).^14,16,21,34,44,46,52^ TSP was predictive in 6/8 (75%) studies.^16,21,34,44,46,52^

Petersen et al.,^44^ demonstrated that high levels of preoperative mTSP assessed in combination with preoperative pain intensity were predictive of 12 months postoperative pain intensity (assessed as the worst pain within the past 24 hours). Petersen et al.,^46^ demonstrated that high levels of mTSP assessed in combination with lower levels of HPT, WDT, and lower Kellgren and Lawrence scores were predictive of 12 months postoperative pain scores (assessed as the worst pain within the past 24 hours). Rice et al.,^52^ reported that preoperative pain intensity, mTSP, trait anxiety, and expected pain predicted 6 months postoperative WOMAC with a specificity of 64% and a sensitivity of 72%. Of note, the Rice et al., study^52^ did not find any significant preoperative predictors for 12 months postoperative WOMAC scores. Kurien et al.,^34^ assessed preoperative TSP_cuff_ and mTSP and found that higher levels of mTSP was associated with the 6-month postoperative pain intensity. Bruehl et al.,^16^ demonstrated that high levels of preoperative mTSP were correlated with higher 6-month postoperative complex regional pain syndrome severity scores (CSS). Edwards et al.,^21^ demonstrated that higher preoperative mTSP, in combination with agreeableness, was predictive of higher 6-month postoperative brief pain inventory (BPI) and WOMAC scores.

#### 3.2.5. Conditioned pain modulation

Conditioned pain modulation was reported as a QST parameter in 9/16 studies (56%).^14,18,21,34,35,44,45,52,58^

A wide variety of different test and conditioning protocols was identified, including the use of PPT as the test stimulus with cold water immersion being the conditioning stimulus (CPM_PPT + cold_),^21,44,52^ PPT and cuff algometry as the test stimulus and cold water immersion as the conditioning stimulus (CPM_cuff + cold_),^58^ PPT and interdigital pinching as test and conditioning stimulus, respectively (CPM_PPT + i.dig.pincing_),^14^ cuff algometry as test and conditioning stimulus (CPM_cuff + cuff_),^15,34,45^ and contact heat as test stimulus with conditioning stimulus hot water (CPM_heat + hot water_).^18^ Conditioned pain modulation predicted postoperative pain in 3/9 (33%) studies.^18,35,58^

Vaegter et al.,^58^ assessed preoperative CPM_PPT + cold_ and found that an impaired CPM-effect was associated with 6-month postoperative pain intensities. Larsen et al.,^35^ assessed preoperative CPM_cuff + cuff_ and found that impaired CPM was associated with 12 months postoperative pain scores. Dürstler et al., 2021 assessed preoperative CPM_heat + hot water_ and found that lower preoperative impaired CPM predicted the presence of 6 months postoperative pain.

#### 3.2.6. Exercise-induced hypoalgesia

Exercise-induced hypoalgesia (EIH) was reported in 1/16 studies (6%),^58^ and an impaired EIH-effect was associated with 6 months postoperative pain relief.

### 3.3. Pharmacological therapy studies

#### 3.3.1. Pressure stimuli

Pressure stimuli were reported in 4/5 studies (80%),^5,20,47,50^ and one study (25%)^50^ found pressure stimuli to be predictive for an analgesic response. Two studies reported on handheld pressure algometry,^5,20^ and one study reported on cuff pressure pain and tolerance thresholds.^47^

Petersen et al.,^50^ found that lower pretreatment PTTs were predictive of a higher analgesic effect of 18-week oral duloxetine treatment.

#### 3.3.2. Temporal summation of pain

Temporal summation of pain was reported in 4/5 studies (80%)^5,20,47,50^ and found predictive of the analgesic effect in 3/4 studies (75%). Arendt-Nielsen et al.,^5^ found that high TSP predicted the nonresponse of 4 weeks of oral COX-2 treatment. Petersen et al.,^47^ found that higher TSP_cuff_ predicted a nonresponse to 3 weeks of oral nonselective NSAIDs and paracetamol. Edwards et al.,^20^ reported that mTSP did not predict the analgesic response of 4 weeks of topical NSAID treatment. Petersen et al.,^50^ demonstrated that higher pretreatment TSP_cuff_ predicted a higher analgesic effect of 18 weeks of oral duloxetine treatment.

#### 3.3.3. Conditioned pain modulation

Conditioned pain modulation was reported in 3/5 studies (60%)^20,48,50^ and found predictive of the analgesic effect in 2/3 studies (67%). Edwards et al.,^20^ found that an impaired CPM_PPT + cold_ effect predicted poor response to 4 weeks of topical nonselective NSAIDs. Petersen et al.,^48^ found that an impaired CPM_cuff + cuff_ effect predicted a poor response to 3 weeks of oral nonselective NSAID and paracetamol.

#### 3.3.4. Offset analgesia

Offset analgesia was assessed in 1/5 studies (20%),^48^ and no studies found offset analgesia to be predictive of an analgesic response.

### 3.4. Exercise-based therapy studies

#### 3.4.1. Thermal stimuli

Thermal stimuli were reported in 1/4 studies (25%),^41^ and no studies (0%) found thermal stimuli to be predictive for treatment response.

#### 3.4.2. Pressure stimuli

Pressure stimuli were reported in 4/4 studies (100%), and 2 studies (50%) found pressure stimuli to be predictive for treatment response. O'Leary et al.,^41^ found that a combination of lower PPTs assessed at the knee, the tibia, and the contralateral arm predicted a nonresponse to 6 to 8 sessions of exercise-based therapy with an odds ratio (OR) of 0.48 (95% confidence interval [CI]: 0.29–0.81). Hansen et al.,^27^ found that lower PPTs assessed at the knee in combination with lower EIH and higher PainDetect score were associated with less pain relief after 12 sessions of exercise-based therapy. Of note, Henriksen et al.,^28^ did find that a change in cuff PPT from baseline to follow-up was associated (*R*^2^ = 0.35) with a change in KOOS from baseline to follow-up.

#### 3.4.3. Temporal summation of pain

Temporal summation of pain was assessed in 3/4 studies (75%), and one study (33%) found TSP to predict treatment outcome. O'Leary et al.,^41^ found that a combination of increased mTSP assessed at the knee, the tibia, and the contralateral arm was associated with a nonresponse to 6 to 8 sessions of exercise-based therapy with a an OR of 2.00 (95% CI: 1.23–3.27).

#### 3.4.4. Mechanical and vibration detection threshold

O'Leary et al.,^41^ assessed mechanical and vibration detection thresholds and did not find these associated with a nonresponse to 6 to 8 sessions of exercise-based therapy.

#### 3.4.5. Conditioned pain modulation

O'Leary et al.,^41^ assessed CPM and found no association with a nonresponse to 6 to 8 sessions of exercise-based therapy.

#### 3.4.6. Exercise-induced hypoalgesia

Hansen et al.,^27^ assessed EIH using 2 different assessments at the m. quadriceps femoris and m. tibialis anterior and found that lower EIH assessed at the m. quadriceps femoris predicted less pain relief after 12 sessions of exercise-based therapy.

### 3.5. Prediction of quantitative sensory testing parameters for treatment outcomes

#### 3.5.1. Total knee arthroplasty

The outcome parameters reported for the 16 surgical studies included 6 on the WOMAC,^14,21,50,52,59,60^ 7 on a VAS score,^6,34,35,38,44–46^ 3 using a NRS^18,40,58^ (Table 1), 1 using complex regional pain syndrome (CRPS) severity scores (CSS),^16^ and 1 on the BPI.^21^ The number of measured QST tests in each study ranged from 1^6,16,18,60^ to 6.^21,34^ A total of 13/16 studies (81%) reported statistically significant associations between preoperative QST and chronic postoperative pain after total knee arthroplasty.

#### 3.5.2. Pharmacological therapies

Five studies were identified where 3 used different VAS scores,^5,47,48^ 1 used average daily pain intensity,^20^ and 1 study used WOMAC and BP^50^ as the outcome parameters (Table 2). Two studies reported on 3 weeks of oral nonselective NSAID in combination with paracetamol,^47,48^ 1 study reported on topical nonselective NSAIDs,^20^ 1 study reported on an oral COX-2 inhibitory NSAID,^5^ and 1 study reported on 18 weeks of oral duloxetine treatment.^50^ The number of QST modalities range from 2^48^ to 3.^5,20,47^ Five of 5 studies (100%) reported an association between pretreatment QST and analgesic effect to pharmacological therapies. A total of 4/4 studies (100%) reported statistically significant associations between pretreatment QST and the analgesic effect to NSAIDs, and 1/1 (100%) study reported statistically significant association between pretreatment QST and the analgesic effect of duloxetine.

#### 3.5.3. Exercise-based therapies

Four studies were identified with 2 studies^27,41^ using the OMERACT-OARSI responder criteria, 1 study using pain after walking,^6^ and 1 study using the KOOS^28^ as the outcome parameters (Table 2). The number of assessed QST modalities ranged from 1 (Arendt-Nielsen et al., 2018) (Arendt-Nielsen et al., 2018) to 7.^41^ A total of 2/4 studies (50%) reported statistical associations between pretreatment QST and pain outcomes after exercise-based therapies.

### 3.6. Quality assessment

Agreement was reached for 95% of the included articles, and any discrepancies were reviewed by one expert in the field (LAN). Consensus was reached on all parameters after discussion. The quality assessment of the included articles is summarized in Table 3.

**Table 3:** Risk of bias based on the quality in prognostic studies (QUIPS) tool for studies investigating the prognostic value of pain sensory profiles on poor response to standard pain therapies for patients with knee osteoarthritis.

	Study participation	Study attrition	Prognostic factor measurement	Outcome measurement	Study confounding	Statistical analysis and reporting
Surgical studies						
Lundblad et al.^38^	M	H	M	M	H	L
Wylde et al.^59^	M	L	L	L	M	M
Noiseux et al.^40^	M	L	L	L	M	L
Petersen et al.^44^	M	L	L	M	M	L
Wylde et al.^60^	L	M	L	M	L	L
Petersen et al.^43^	L	M	L	M	M	L
Bossmann et al.^14^	M	M	L	L	L	L
Vaegter et al.^58^	M	L	L	L	M	L
Arendt-Nielsen et al.^6^	L	M	L	M	L	M
Petersen et al.^46^	L	M	M	L	M	L
Rice et al.^52^	L	L	L	L	M	L
Kurien et al.^34^	M	L	L	M	M	L
Dürsteler et al.^18^	L	L	L	L	M	M
Larsen et al.^35^	M	L	L	L	M	L
Bruehl et al.^16^	L	M	L	L	M	L
Edwards et al.^21^	M	H	L	L	M	M
Pharmacological studies						
Arendt-Nielsen et al.^5^	L	M	L	L	M	L
Edwards et al.^20^	M	M	L	L	M	L
Petersen et al.^47^	L	M	L	L	M	L
Petersen et al.^48^	L	L	L	L	M	L
Petersen et al.^50^	L	L	L	L	M	L
Exercise-based studies						
Henriksen et al.^28^	L	M	L	M	M	M
O'Leary et al.^41^	L	L	L	L	L	L
Arendt-Nielsen et al.^6^	L	L	M	L	L	M
Hansen et al.^27^	L	L	L	L	M	M

H, high risk of bias; L, low risk of bias; M, medium risk of bias.

### 3.7. Meta-analysis

**Figure 2.**
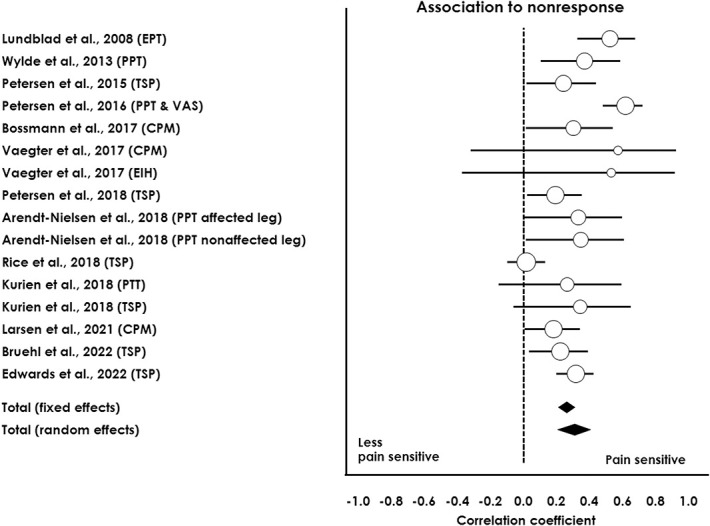
Forest plot from surgical studies assessing associations between preoperative pain sensory profiles and chronic postoperative pain after total knee arthroplasty. The correlation coefficient (positive values) indicates the strength of association between the preoperative QST parameter and the postoperative pain outcome. CPM, conditioned pain modulation; EIH, exercise-induced hypoalgesia; EPT, electrical pain threshold; PPT, pressure pain threshold; PTT, pressure tolerance threshold; QST, quantitative sensory testing; VAS, Visual Analogue Scale.

**Figure 3.**
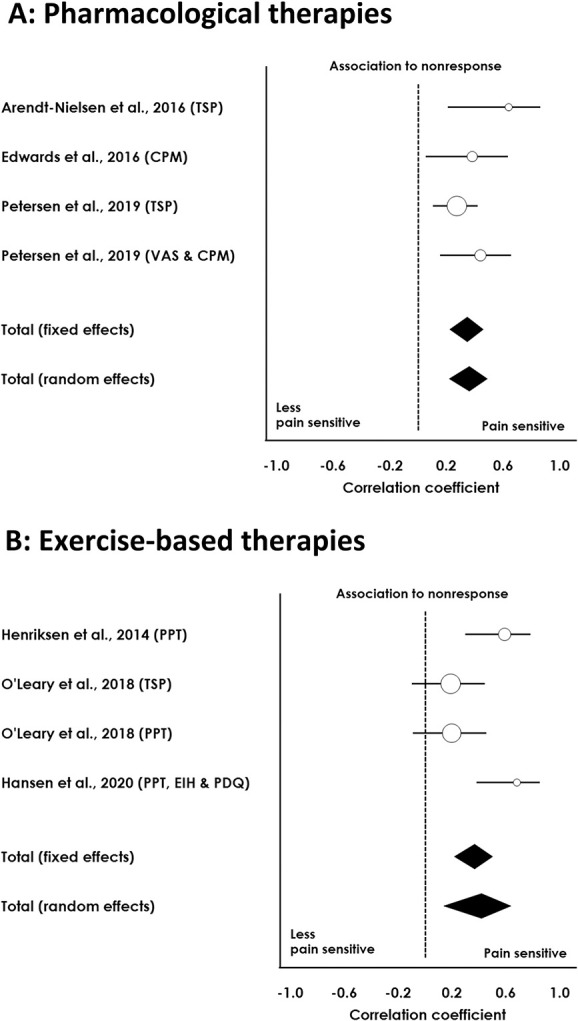
Forest plot from pharmacological (A) and exercise-based (B) therapy studies assessing associations between preoperative pain sensory profiles and response to therapy. The correlation coefficient (positive values) indicates the strength of association between the preoperative QST parameter and the postoperative pain outcome. CPM, conditioned pain modulation; EIH, exercise-induced hypoalgesia; EPT, electrical pain threshold; PPT, pressure pain threshold; PDQ, PainDetect Questionnaire; QST, quantitative sensory testing; VAS, Visual Analogue Scale.

For the surgical studies, a meta-analysis based on 1367 patients indicated that a pronociceptive preoperative profile was associated with higher risk of chronic postoperative pain in both a fixed (*P* < 0.001) and random-effects (*P* < 0.001) model (see Table 4 for meta-analysis and Fig. 2 for forest plot). For the pharmacological studies, a meta-analysis based on 225 patients indicated that a pronociceptive pretreatment QST profile was associated with a less beneficial analgesic response to NSAIDs in a fixed (*P* < 0.001) and random-effects (*P* < 0.001) model (see Table 5 for meta-analysis and Fig. 3 for forest plot). For the exercise-based studies, a meta-analysis based on 154 patients indicated that a pronociceptive pretreatment QST profile was associated with less pain relief after exercise-based therapy for both fixed (*P* < 0.001) and random-effects (*P* = 0.004) models (see Table 5 for meta-analysis and Fig. 3 for forest plot). A meta-analysis for duloxetine was not conducted, since only one study^50^ assessed the predictive value of QST on the analgesic effect of duloxetine for OA pain.

**Table 4:** Weighted correlations with 95% confidence interval for the correlation between preoperative quantitative sensory testing measures and postoperative pain.

Study	Sample size	Correlation coefficient	95% CI	z	*P*	Weight (%)	
						Fixed	Random
Lundblad et al.^38^ (EPT)	69	0.522	0.326 to 0.675			4.91	7.08
Wylde et al.^59^ (PPT)	51	0.370	0.105 to 0.586			3.57	6.28
Petersen et al.^44^ (TSP)	78	0.240	0.0185 to 0.439			5.58	7.38
Petersen et al.^43^ (PPT & VAS)	103	0.616	0.480 to 0.723			7.44	8.00
Bossmann et al.^14^ (CPM)	47	0.300	0.0140 to 0.541			3.27	6.06
Vaegter et al., 2017 (CPM)*	7	0.570	−0.321 to 0.926			0.30	1.13
Vaegter et al.^58^ (EIH)*	7	0.530	−0.371 to 0.917			0.30	1.13
Petersen et al.^46^ (TSP)	130	0.193	0.0215 to 0.353			9.45	8.46
Arendt-Nielsen et al., 2018 (PPT affected leg)*	35	0.330	−0.00365 to 0.598			2.38	5.21
Arendt-Nielsen et al.^6^ (PPT nonaffected leg)*	35	0.345	0.0133 to 0.608			2.38	5.21
Rice et al.^52^ (TSP)	291	0.0160	−0.0992 to 0.131			21.43	9.58
Kurien et al., 2018 (PTT)*†	25	0.262	−0.149 to 0.595			1.64	4.22
Kurien et al.^34^ (TSP)*	25	0.343	−0.0603 to 0.650			1.64	4.22
Larsen et al.^35^ (CPM)†	131	0.180	0.00874 to 0.341			9.52	8.47
Bruehl et al.^16^ (TSP)	110	0.220	0.0342 to 0.391			7.96	8.14
Edwards et al.^21^ (TSP)	248	0.316	0.199 to 0.424			18.23	9.41
Total (fixed effects)	1392	0.261	0.210 to 0.310	9.785	<0.001	100.00	100.00
Total (random effects)	1392	0.309	0.206 to 0.405	5.670	<0.001	100.00	100.00

The table demonstrates both fixed and random effects based on weighted fixed and random-effects correlation coefficients (Fisher z transformation).

* Sample size halved per guidelines, to account for multiple inclusions.

† Originally a negative correlation but reversed so that a larger positive value indicates higher pain sensitization.

95% CI, 95% confidence interval; CPM, conditioned pain modulation; EIH, exercise-induced hypoalgesia; EPT, electrical pain threshold; PPT, pressure pain threshold; PTT, pressure tolerance threshold; VAS, Visual Analogue Scale.

**Table 5:** Weighted correlations with 95% confidence interval for the correlation between pretreatment pain sensory profiles and analgesic effect in pharmacological and exercise-based therapy studies.

Study	Sample size	Correlation coefficient	95% CI	z	*P*	Weight (%)	
						Fixed	Random
Pharmacological therapies							
Arendt-Nielsen et al.^5^ (TSP)	16	0.639	0.210 to 0.862			6.10	7.71
Edwards et al.^20^ (CPM)	35	0.380	0.0535 to 0.633			15.02	17.73
Petersen et al.^47^ (TSP)	132	0.270	0.104 to 0.421			60.56	53.46
Petersen et al.^48^ (VAS & CPM)	42	0.440	0.157 to 0.656			18.31	21.10
Total (fixed effects)	225	0.346	0.222 to 0.458	5.260	<0.001	100.00	100.00
Total (random effects)	225	0.360	0.219 to 0.487	4.733	<0.001	100.00	100.00
Exercise-based therapies							
Henriksen et al.^28^ (PPT)	31	0.590	0.298 to 0.781			19.72	23.71
O'Leary et al.^41^ (TSP)*	50	0.188	−0.0953 to 0.443			33.10	27.59
O'Leary et al.^41^ (PPT)*†	49	0.198	−0.0881 to 0.454			32.39	27.45
Hansen et al.^27^ (PPT, EIH & PDQ)	24	0.680	0.381 to 0.850			14.79	21.25
Total (fixed effects)	154	0.366	0.216 to 0.500	4.578	<0.001	100.00	100.00
Total (random effects)	154	0.417	0.138 to 0.635	2.849	0.004	100.00	100.00

The table demonstrates both fixed and random effects based on weighted fixed and random-effects correlation coefficients (Fisher z transformation).

* Sample size halved per guidelines, to account for multiple inclusions.

† Originally a negative correlation but reversed so that a larger positive value indicates higher pain sensitization.

95% CI, 95% confidence interval; CPM, conditioned pain modulation; EIH, exercise-induced hypoalgesia; EPT, electrical pain threshold; PPT, pressure pain threshold; PDQ, PainDetect Questionnaire; VAS, Visual Analogue Scale.

Heterogeneity for the surgical meta-analysis was substantial (I^2^ = 70.05%), and the Eggers test was nonsignificant (*P* = 0.117), indicating no evidence of publication bias. Low heterogeneity was observed for the pharmaceutical meta-analysis (I^2^ = 13.74%), and the Eggers test was significant (*P* = 0.00389), suggesting possible publication bias. Finally, heterogeneity for the exercise-based meta-analysis was substantial (I^2^ = 69.65%), and the Eggers test was significant (*P* = 0.042), suggesting a potential publication bias.

## 4. Discussion

The current systematic review and meta-analysis describe the predictive role of QST profiling for pain outcomes after total knee arthroplasty, NSAID, and duloxetine therapy and exercise-based therapy in patients with osteoarthritis. The systematic review identified that 13/16 studies (81%) reported an association between preoperative QST profiling and chronic postoperative pain, 5/5 studies (100%) reported an association between pretreatment QST profiling and analgesic effects of NSAIDs and duloxetine, and 2/4 studies (50%) reported an association between pretreatment QST profiling and response to exercise-based therapy. The meta-analyses indicated that QST parameters were associated with poor outcome after TKA surgery, and NSAID and exercise-based therapies, which suggests that patients with a pan sensitive profile are less likely to respond to these standard pain treatments for osteoarthritic pain.

### 4.1. The predictive value of quantitative sensory testing profiling

Other systematic reviews have reported a possible predictive value of QST for postoperative pain after different surgical interventions.^49,54^ One review included acute and chronic postoperative pain measures and concluded that QST before TKA surgery did not consistently predict pain after surgery,^54^ whereas a recent review^49^ found that preoperative QST was predictive in 68% of cases for chronic postoperative pain after different surgical procedures. This may indicate that preoperative QST is a better or at least more consistent predictor of chronic postoperative pain than acute postoperative pain as previously suggested.^54^ The current systematic review is the first to review the predictive value of QST for responses to pharmacological-based and exercise-based therapies in patients with OA.

The systematic review demonstrated that widespread pressure hyperalgesia (25%), TSP (38%), and CPM (19%) were the most frequently reported predictive QST parameters for continued pain after total knee arthroplasty, which is consistent with earlier reviews on multiple different surgical interventions.^49,54^ In addition, TSP (60%) and CPM (40%) were the most frequently reported predictive QST parameters for the pharmacological treatments. Finally, widespread pressure pain hyperalgesia (50%) was the most frequently reported predictive QST parameter for pain relief after exercise-based therapy. The results from the meta-analyses indicate that pain-sensitive pretreatment QST profiles are associated with less pain relief after total knee arthroplasty, NSAIDs, and exercise-based therapies. Conclusively, these findings suggest that knee OA patients defined as “sensitized” might not respond sufficiently to standard therapies as recommended by the OARSI guidelines.^65^

### 4.2. Quality assessment

The risk of bias analysis identified low-to-moderate bias distributed among the 6 categories: study participation, study attrition, prognostic factor of measurement, outcome measurement, confounding, and statistical analysis/reporting. The study participation was mainly biased due to missing data on sampling frame, recruitment, and place for assessment, whereas the study attrition often lacked information on missing data, loss to follow-up, and differences in the patients who completed the study and those who did not. The main reasons for bias in the prognostic factor of measurement and outcome categories were related to the validity or reliability of the measures (both prognostic and outcome). The confounders category revealed moderate bias due to the lack of clear definition of confounding variables and attempts to account for these in the study designs. Statistical analyses and reporting were not consistent in the included articles.

### 4.3. Future perspectives

The current systematic review and meta-analysis indicates that a subset of OA patients exist, who are not responding adequately to the 3 investigated standard pain treatments for OA, and it is, therefore, important to consider alternative treatment options for these patients.

Animal studies indicate that serotonin and noradrenaline are important neurotransmitters for functional descending pain inhibitory control,^8,37^ which is assessed using the CPM paradigm in humans.^64^ The OARSI recommends duloxetine (a serotoninnoradrenalin reuptake inhibitor) as a pharmaceutical treatment for pain in OA when depression and widespread pain is present.^9^ Four weeks of duloxetine have been found to provide pain relief and restore impaired CPM in patients with painful diabetic neuropathies, indicating a link between modulation of serotonin and noradrenalin and QST response.^63^ Recently, Koh et al.,^32^ randomized pain sensitive patients to duloxetine or placebo before and 6 weeks after total knee arthroplasty and found significant 12 weeks postoperative pain relief in the duloxetine group when compared with placebo. In addition, administration of ketamine (an N-methyl-d-aspartate receptor antagonist) to patients with fibromyalgia can reduce TSP,^26^ and since facilitated TSP is associated with chronic postoperative pain after total knee arthroplasty,^1,45,46^ this may be a future target for patients undergoing surgery. Based on these findings, it may be hypothesized that pain-sensitive patients (based on TSP and CPM) before total knee arthroplasty could benefit from preoperative pharmaceutical interventions to normalize the facilitation of pain mechanisms, and large scale studies are initiated to pursue this hypothesis.^56^

It is important to acknowledge that several factors interact with the pain sensory profiles,^43^ and multiple other preoperative risk factors are associated to poor response to standard pain therapies. Cognitive factors (such as anxiety, depression, or pain catastrophizing) are a well-known risk factor for chronic postoperative pain.^19,51^ A recent study demonstrated that the combination of preoperative CPM and pain catastrophizing yielded a stronger prediction model than each of the assessments alone,^35^ which suggests that adding cognitive factors to the existing prediction models will strength the models in the future. In addition, quality of sleep is decreased in different chronic pain conditions, including OA,^15^ and this have been shown to further deteriorate and increase, eg, cognitive factors^15^ and inflammation,^30^ respectively. Elevated levels of proinflammatory cytokines have been associated to pain after total knee arthroplasty,^24^ and proinflammatory cytokines can sensitize peripherally and centrally pain pathways,^55^ thereby potentially yielding a pronociceptive profile. It is, therefore, highly likely that a combination of QST and inflammatory mediator assessments will increase the predictive value for chronic postoperative pain after total knee arthroplasty in the future.

Likely, many complex interactions between risk factors for a poor response to standard OA pain therapies exist, and understanding these interactions in the future is likely to advance the field towards a personalized mechanistic-based treatment approach for OA.

### 4.4. Methodological considerations and limitations for the interpretation of the review

The search strategy was limited to 2 databases and English language, and it cannot be excluded that pertinent papers were missed. The included studies used a wide variety of different assessment methods, number of included patients, and outcome measures, which should be considered when assessing this work.

The studies included in this systematic review and meta-analysis displayed a medium-to-large degree of heterogeneity for the surgical, pharmacological, and exercise-based therapy studies, which traditionally will complicate the conduct of a meta-analysis. The current work did present a meta-analysis based on recalculations of odds ratios and *R*^2^ values which may introduce interpretative limitations which should be considered. In addition, the number of studies included in the meta-analysis predominantly favored associations between QST and treatment outcomes, which likely leads to publication bias, and this should be considered when interpreting the results.

The pharmacological and exercise-based meta-analysis is linked underpowered for a meta-analysis. The surgical, pharmacological, and exercise-based meta-analyses did indicate a trend towards publication bias, which should be considered when interpreting the data.

Only a single study was found exploring the predictive value of QST for the analgesic effect of duloxetine, and therefore, a meta-analysis was not conducted. An updated systematic review and meta-analysis should be completed when sufficient data are available.

The current work combined QST profiles into “pronociceptive” and “antinociceptive” profiles due to the lack of studies on individual QST methodologies. Ideally, a meta-analysis should be conducted on single parameters, and this should be considered when interpreting the results of the current work.

## 5. Conclusion

This systematic review and meta-analysis identified 13/16 surgical (81%), 5/5 pharmaceutical (100%), and 2/4 (50%) nonsurgical and nonpharmaceutical studies that reported a statistically significant association between pretreatment QST parameters and pain responses after treatment for patients with knee osteoarthritis. Three meta-analyses demonstrated that a pretreatment QST profiling to some degree could predict poor pain-relieving response after total knee arthroplasty, NSAIDs, and exercise-based therapy. Pretreatment pressure pain thresholds, temporal summation of pain, and conditioned pain modulation were the most frequently reported QST predictors. The studies included in the meta-analyses suffered from substantial risk of publication bias, which should be considered when interpreting the results.

Based on this work, it is hypothesized that a subset of specific pain-sensitive patients with osteoarthritis exist and that these patients do not respond adequately to standard osteoarthritic pain treatments. Research should focus on to identify this group and offer a more comprehensive pain management program.

## Disclosures

The authors have no conflict of interest to declare.

## Supplementary Material

SUPPLEMENTARY MATERIAL

## Appendix A. Supplemental digital content

Supplemental digital content associated with this article can be found online at http://links.lww.com/PR9/A194.
