# Supplementary material for: Quantitative sensory testing as an assessment tool to predict the response to standard pain treatment in knee osteoarthritis: a systematic review and meta-analysis
Source: Pain Rep. 2023 Jun 5;8(4):e1079. doi: 10.1097/PR9.0000000000001079 (PMC11065125; doi:10.1097/PR9.0000000000001079)
Supplement: SUPPLEMENTARY MATERIAL [file painreports-8-e1079-s001.pdf]

Supplementary tables

	FOCUS	Example key words (MEDLINE example)
1	Osteoarthritis	Osteoarthritis[MeSH Terms]
2	Quantitative sensory testing	Quantitative sensory testing[tw]; QST[tw]
3	Central pain mechanisms	Conditioned pain modulation[tw]; temporal summation of pain[tw]; CPM[tw]; TSP[tw]
4	Surgery	Postoperative pain[MeSH Term]; Postsurgical pain[tw]
5	Analgesic effect (pharmaceutical)	Analgesia[tw]; drugs[tw]; Drug therapy[tw]
6	Exercise	Exercise[MeSH Term]; Exercise therapy[tw]; physical therapy[tw]
7	Limits (time)	2000:2023[pdat]

Supplementary table 1. The MeSH and textword strings used and permuted for Medline database.

**Search strings for PUBMED**
#1<br>((((((Osteoarthritis[MeSH Major Topic]) AND (Quantitative sensory testing[Text Word])) OR (QST[Text Word])) OR (Conditioned pain modulation[Text Word])) OR (CPM[Text Word])) OR (temporal summation[Text Word])) OR (TSP[Text Word]);PDAT:2000-2023
#2<br>((Analgesia[Text Word]) OR (Drugs[Text Word])) OR (Drug therapy[Text Word]);PDAT:2000-2023
#3<br>Exercise[MeSH Terms];PDAT:2000-2023
#4<br>(Postoperative pain[MeSH Terms]) OR (Postsurgical pain[Text Word]);PDAT:2000-2023
#1 AND #2
#1 AND #3
#1 AND #4
#1 AND #2 OR #3 OR #4

Supplementary table 2: Example of search strategy for PubMed database.
